# Effect of Electroacupuncture at Zusanli (ST36) on Sepsis Induced by Cecal Ligation Puncture and Its Relevance to Spleen

**DOI:** 10.1155/2020/1914031

**Published:** 2020-10-07

**Authors:** Dong-ping Xie, Geng-biao Zhou, Rui-lan Chen, Xiao-lian Qin, Jiong-dong Du, Yan Zhang, Yan-na Weng, Shu-tao Mai, Fang Lai, Yun Han

**Affiliations:** ^1^The Second Affiliated Hospital of Guangzhou University of Chinese Medicine, Guangzhou, Guangdong, China; ^2^Guangdong Provincial Hospital of Chinese Medicine, Guangzhou, Guangdong, China; ^3^Guangdong Provincial Key Laboratory of Research on Emergency in TCM, Guangzhou, Guangdong, China; ^4^Guangdong Second Traditional Chinese Medicine Hospital, Guangzhou, Guangdong, China; ^5^Chao En-Xiang Famous Chinese Medicine Expert Inheritance Studio, Guangdong Provincial Hospital of Chinese Medicine, Guangzhou, Guangdong, China

## Abstract

**Background:**

Acupuncture at Zusanli (ST36), Quchi (LI11), and Tianshu (ST25) is commonly used in septic patients by traditional Chinese physicians. The protective effect of acupuncture at ST36 on the intestinal barrier is associated with Cholinergic Anti-Inflammatory Pathway (CAIP). However, its detailed mechanism and whether acupuncture at LI11 and ST25 have similar effects to ST36 remain unclear.

**Aim:**

To explore the effects of electroacupuncture (EA) at ST36, LI11, and ST25 on septic rats and investigate the role of the spleen in the treatment of EA at ST36.

**Methods:**

A septic rat model caused by cecal ligation and puncture (CLP) and a postsplenectomy (SPX) CLP rat model were established. Rats were divided into nine groups depending on different treatments. Serum levels of TNF-*α*, IL-10, D-lactic acidosis (D-LA), double amine oxidase (DAO), and T-lymphocyte subgroup level in intestinal lymph nodes were compared.

**Results:**

EA could not improve the 2-day survival of CLP rats. For CLP rats, EA at ST36 and LI11 significantly decreased the levels of TNF-*α*, IL-10, DAO, and D-LA in serum and normalized intestinal T-cell immunity. For SPX CLP rats, EA at ST36 failed to reduce serum concentrations of TNF-*α*, IL-10, and D-LA but increased the values of CD3^+^CD4^+^/CD3^+^CD8^+^ cells and Treg/Th17 cells.

**Conclusions:**

EA at ST36 and LI11, respectively, could alleviate inflammation reaction, protect the intestinal barrier, and maintain intestinal T-cell function in septic rats. Spleen participated in the protective effect of EA at ST36 in sepsis.

## 1. Introduction

Around the world, sepsis has high incidence (about 31.5 million per year), which is even higher in intensive care units, and high mortality (more than 5.3 million people per year), leading to the heavy financial burden (more than 14.6 billion dollars per year) [1, 2]. Sepsis 3.0 declared that the host's dysregulated response to infection is a strong trigger to life-threatening organ dysfunction [3]. It reminds us that, apart from anti-infection, immunoregulation is also an important strategy to maintain the host's organ function in sepsis.

It is well known that intestinal barrier dysfunction is a result and a cause of sepsis development. At the beginning of sepsis, a cytokines storm is triggered by bacteria and their products and then drives into local intestinal and systemic inflammation, in which cells like epithelial cells and CD3^+^CD4^+^ T-cell apoptosis increase, leading to high permeability, hypoperfusion, bacterial translocation, and microbiome shifts in the gut [4]. Serum level of proinflammation cytokines, intestinal permeability, and T lymphocyte compositions in intestinal lymph nodes are indexes for intestinal function evaluation. They have an impact on each other in the pathophysiologic process of sepsis. D-lactic acidosis (D-LA) and double amine oxidase (DAO) are valid indicators for intestinal permeability. They are scarcely detected in blood circulation normally but increase sharply in serum when the intestinal barrier is damaged in sepsis [5, 6].

In China, acupuncture is often used in patients with gastrointestinal dysfunction, in which Zusanli (ST36), Quchi (LI11), and Tianshu (ST25) are acupoints frequently chosen [7–9]. The mechanism of electroacupuncture (EA) at ST36 protecting the host's intestinal function from several diseases including sepsis is reported to be associated with the Cholinergic Anti-Inflammatory Pathway (CAIP) [7, 10–15]. After activating the CAIP by peripheral or central stimuli, acetylcholine is released and binds to *α*7 nicotinic acetylcholine receptor (*α*7nAchR), stimulating T cells to secrete noradrenaline, and finally acts on macrophages to suppress the production of inflammatory cytokines [16–19]. The spleen was regarded as an essential organ for CAIP, for cytokine production was dramatically suppressed after splenectomy [20, 21]. However, researchers found that there is no innervation from the vagus to the spleen [22–25]. Focusing on the possible mediators, among which choline acetyltransferase positive T-lymphocytes and mesothelial cells were strong candidates, several models of CAIP were proposed [18, 19]. Given the special role of the spleen in CAIP, we aimed to clarify whether it took part in the protective effect of EA at ST36 on sepsis with a postsplenectomy (SPX) septic rat model induced by cecal ligation and puncture (CLP). Besides, we also investigated the effects of EA at LI11 and at ST25 on sepsis in this study.

## 2. Materials and Methods

### 2.1. Animals

All experimental protocols were approved by the Ethics Committee for Animal Experiment of Guangdong Provincial Hospital of Chinese Medicine (permit number: 2015007) and carried out in accordance with the Guide for the Care and Use of Laboratory Animals of the National Institutes of Health [26]. Pathogen-free male Sprague-Dawley rats, weighing 240–270 g, were purchased from the Experimental Animal Center of Guangdong Province. All rats used in this study were housed under specific pathogen-free conditions (12-hour light/dark cycle, 22–24°C, and 60–70% humidity) and were fed with standard rodent diet and water daily.

### 2.2. Experimental Design

To determine the effect of EA and spleen on treating sepsis, we assigned rats randomly to groups listed as follows: Sham group (*n* = 6), CLP group (*n* = 12), CLP + Zusanli group (*n* = 12), CLP + Quchi group (*n* = 12), CLP + Tianshu group (*n* = 12), CLP + GTS21 (a specific agonist of *α*7nAchR) group (*n* = 12), SPX + CLP group (*n* = 12), SPX + CLP + Zusanli group (*n* = 12), and SPX + CLP + GTS21 group (*n* = 12). A two-day intervention of EA or GTS21 was given to CLP rats in CLP + Zusanli group, CLP + Quchi group, CLP + Tianshu group, CLP + GTS21 group, SPX + CLP + Zusanli group, and SPX + CLP + GTS21 group. Then, rats were sacrificed 48 hours after the surgery of CLP.

### 2.3. Construction of Rat Models

3% sodium pentobarbital (30 mg/kg body weight) was intraperitoneally injected into rats before a surgical operation. After anesthetization, the abdominal area was shaved and disinfected.

A sepsis rat model induced by CLP was prepared following a previous study [27]. After a middle laparotomy, we found out the cecum and performed the CLP surgery as below to get mortality of about 50%. We ligated the rat's cecum at 75% position of its length (from the end of the cecum to the initial part of ascending colon), punctured twice throughout its gut wall with a 22-gauge needle carefully to avoid puncturing the blood vessels. Then, a small amount of cecal content was gently extruded out of the cecum, and the abdominal cavity was closed after careful suture of abdominal musculature and skin. In the Sham group, rats' abdominal cavities were closed without ligation and puncture after the cecum was found.

In order to explore whether the therapeutic effect of EA at ST36 is spleen-dependent, splenectomy was performed as below to rats one week before CLP. After an overnight fast of 12 hours, rats were anesthetized for skin preparation, disinfection, and abdominal operation. Then, we opened the abdominal cavity along the left costal margin, identified the spleen, put aside its stomach and bowel, and dissociated surrounding tissues so that the spleen was exposed sufficiently. Immediately afterward, vessel disconnection around the spleen was performed with vessel clamp, No.0 operation silk thread, and scissors. Finally, we removed the spleen and sutured the abdominal musculature and skin [28].

### 2.4. Electroacupuncture at the Three Acupoints and GTS21 Administration

After CLP operation, a two-day EA treatment or GTS21 administration was performed once a day. For EA treatment, after determining the localization of certain acupoints (Zusanli-ST36, Quchi-LI11, and Tianshu-ST25), rats were acupunctured with a 0.35 mm diameter needle without any hand-technique like lifting, thrusting, and rotating. Then, a stimulation at an intensity of 2 V (load voltage) and a frequency of 3 Hz was given for 15 minutes using the Electronic Acupuncture Treatment Instrument (96805- I Type, Xinsheng Industrial Limited Liability Company, Qingdao, China). For GTS21 (a specific agonist of *α*7nAchR; ab120560, Abcam, England) administration, liquid medicine was injected to CLP rats intraperitoneally with a dose of 4 mg/kg/d for two days [28].

### 2.5. Euthanasia Methods

Rats intended for euthanasia were first anesthetized with an intraperitoneal dose of 3% pentobarbital (30 mg/kg body weight), and then, a 4 cm U-shaped abdominal incision was performed after shaving and disinfection. After exposing the abdominal aorta, we collected blood samples by inserting the blood taking needles until they bled to death.

### 2.6. Enzyme-Linked Immunosorbent Assay

The blood sample was harvested from the abdominal aorta and centrifuged (1,600 g for 10 minutes at 4°C) for serum. The serum was stored at −80°C until thawing at the time of assay. Following the protocol of highly sensitive enzyme-linked immunosorbent kits (Dakewe Biotech Limited Liability Company, Shenzhen, China), we measured the concentrations of TNF-*α* (art. number: 1317202) and IL-10 (art. number: 1311002).

### 2.7. Detection of DAO and D-LA

We detected serum levels of D-LA and DAO in this study using biochemical kits bought from Cusabio Technology Limited Liability Company in Wuhan, China. Following the instruction, blood samples from every rat were centrifuged as mentioned above for supernatant. The optical density value of each supernatant and standard substances was measured with a microplate reader at 450 nm within 30 min. Concentrations of DAO and D-LA were estimated using standard curves.

### 2.8. Flow Cytometry

Mesenteric lymph nodes were isolated from each animal and cut into smaller pieces of approximately 1 mm^2^. After washing with phosphate buffer saline, the tissue was disrupted mechanically into smaller pieces. The remaining tissue aggregates were removed by a 70 *μ*m nylon cell strainer. The resulting cell suspension was centrifuged twice at 1,000 rpm, for 5 min at 4°C in a 30% isotonic Percoll solution (Borunlaite Sci & Tech Co., Ltd., Beijing, China). The supernatant containing epithelial cells and debris was discarded; the cell pellet was washed and resuspended in RPMI1640 medium. Samples were stained with anti-CD3 PE (12-0030-82, eBioscience, San Diego, Calif), anti-CD4 FITC (11-0040-82, eBioscience, San Diego, Calif), anti-CD8a FITC (11-0084-82, eBioscience, San Diego, Calif), anti-CD25 PE (12-0390-82, eBioscience, San Diego, Calif), anti-IL17RB (BS-2610R, Bioss, Beijing, China), and anti-Foxp3 eFluor 660 (50-5773-82, eBioscience, San Diego, Calif). Flow cytometry data were acquired using a FACSCalibur (BD Biosciences, San Diego, CA) and analyzed with FlowJo software (Tree Star, Ashland, OR).

### 2.9. Statistical Analysis

Data analysis was performed with the SPSS software (version 17.0, Chicago, IL, USA). Enumeration data were analyzed by Pearson's *X*^2^ test. Continuous variables were expressed as means ± standard deviation(M ± SD), and differences among experimental groups were compared using analysis of variance (one-way ANOVA) followed by Tukey's post hoc test. *P* < 0.05 was considered to be statistically significant.

## 3. Results

### 3.1. EA at ST36, LI11, and ST25 Cannot Lower the 2-Day Mortality of CLP Rats

During the observation period, no rats died in the Sham group. As shown in Table 1, the mortalities of CLP rats ranged from 41.67% to 58.33% in different groups, but no significant differences were found among them. It indicated that EA at each of the three acupoints (ST36, LI11, and ST25) as well as administration of GTS21 had no impact on the CLP rats' mortality.

### 3.2. Both EA at ST36 and EA at LI11 Can Reduce Systemic Inflammation Induced by CLP

**Figure 1:**
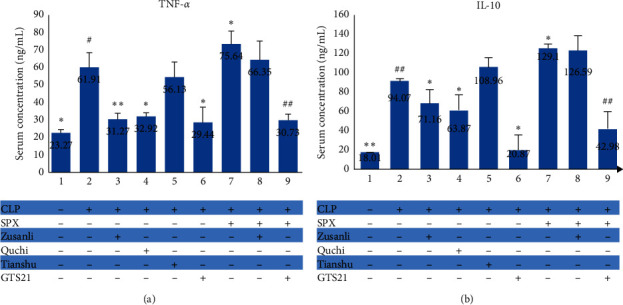
Serum levels of TNF-*α* (a) and IL-10 (b) in each group. ^*∗*^ − *P* < 0.05 vs. CLP group; ^*∗∗*^ − *P* < 0.01 vs. CLP group; ^#^ − *P* < 0.05 vs. SPX + CLP group; ^##^ − *P* < 0.01 vs. SPX + CLP group.

To evaluate whether EA at one of the three acupoints had an effect on host systemic inflammatory reaction, serum levels of TNF-*α* and IL-10 were compared among the groups of Sham, CLP, CLP + Zusanli, CLP + Quchi, CLP + Tianshu, and CLP + GTS21. The results showed significant differences in both TNF-*α* (*F* = 37.17, *P* < 0.01) and IL-10 (*F* = 58.76, *P* < 0.01) expressions among all groups. Compared with the CLP group, all intervention groups except the CLP + Tianshu group (*P* > 0.05) expressed lower levels of TNF-*α* (Figure 1(a)) and IL-10 (Figure 1(b)). However, neither the CLP + Zusanli group (*P*=0.997) nor the CLP + Quchi group (*P*=0.960) had different levels of TNF-*α* compared with the CLP + GTS21 group. These results indicated that EA at ST36 and at LI11 could alleviate septic rat's systemic inflammation to an extent comparable to GTS21 administration.

### 3.3. Both EA at ST36 and EA at LI11 Lessen Rat's Intestinal Permeability Enhanced by Sepsis

**Figure 2:**
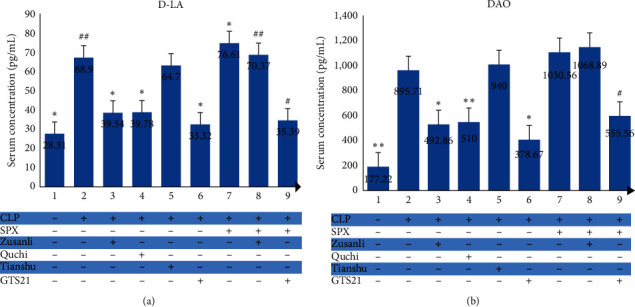
Serum concentrations of DAO and D-LA in each group. ^*∗*^ − *P* < 0.05 vs. CLP group; ^*∗∗*^ − *P* < 0.01 vs. CLP group; ^#^ − *P* < 0.05 vs. SPX + CLP group; ^##^ − *P* < 0.01 vs. SPX + CLP group.

We compared serum concentrations of D-LA and DAO among the groups of Sham, CLP, CLP + Zusanli, CLP + Quchi, CLP + Tianshu, and CLP + GTS21 and found significant differences (DAO : *F* = 89.36, *P* < 0.01; D-LA : *F* = 93.68, *P* < 0.01). As shown in Figure 2, rats had an obvious increase in DAO (177.22 ± 34.35 vs. 895.71 ± 126.66 pg/mL, *P* < 0.01, Figure 2(a)) and D-LA (28.31 ± 3.63 vs. 68.90 ± 3.56 pg/mL, *P* < 0.01, Figure 2(b)) secretion after CLP. As expected, the administration of GTS21 to rats after CLP could bring a drop to both DAO and D-LA levels. Although EA at ST25 had no impact on DAO and D-LA reduction in CLP rats, it is exciting that both rats in the CLP + Zusanli group and CLP + Quchi group had a lower concentration of DAO and D-LA than that in the CLP group. But no statistical difference in DAO (CLP + Zusanli vs. CLP + GTS21 : *P*=0.174; CLP + Quchi vs. CLP + GTS21 : *P*=0.128) and D-LA (CLP + Zusanli vs. CLP + GTS21 : *P*=0.189; CLP + Quchi vs. CLP + GTS21 : *P*=0.225) levels was found compared with the CLP + GTS21 group, respectively. These results suggested that EA at ST36 or EA at LI11 had a similar beneficial effect on the intestinal barrier protection to CAIP agonist GTS21 treatment, while EA at ST25 showed no remarkable effect on intestinal permeability in this CLP rat model.

### 3.4. EA at ST36, LI11, and ST25, Respectively, Was Helpful for Normalizing Dysfunction of Intestinal Immunity in CLP Rats

**Figure 3:**
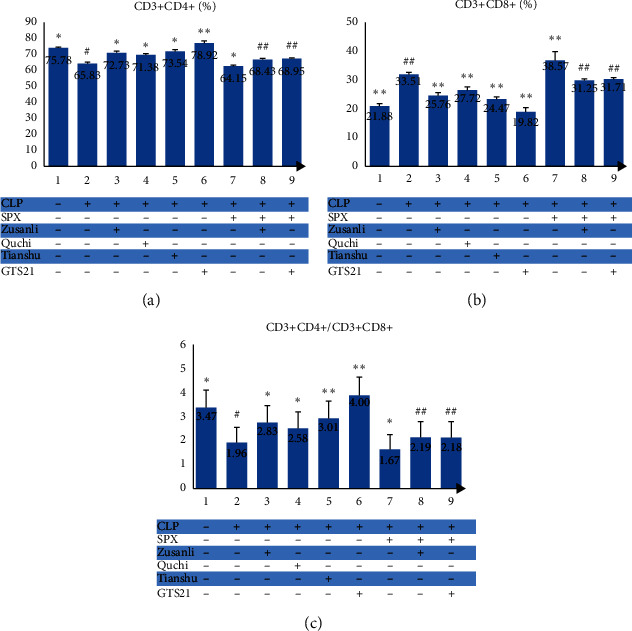
CD3^+^CD4^+^ (%), CD3^+^CD8^+^ (%), and CD3^+^CD4^+^/CD3^+^CD8^+^ in each group. ^*∗*^ − *P* < 0.05 vs. CLP group; ^*∗∗*^ − *P* < 0.01 vs. CLP group; ^#^ − *P* < 0.05 vs. SPX + CLP group; ^##^ − *P* < 0.01 vs. SPX + CLP group.

**Figure 4:**
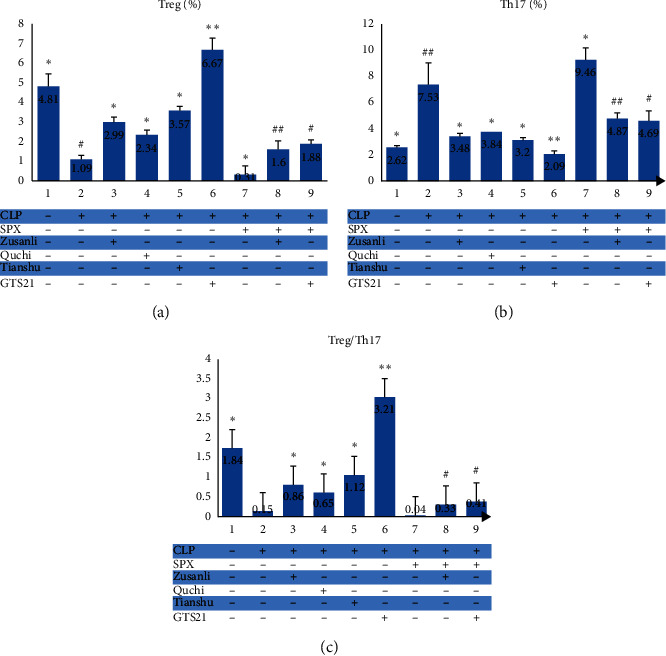
Treg (%), Th17 (%), and Treg/Th17 in each group. ^*∗*^ − *P* < 0.05 vs. CLP group; ^*∗∗*^ − *P* < 0.01 vs. CLP group; ^#^ − *P* < 0.05 vs. SPX + CLP group; ^##^ − *P* < 0.01 vs. SPX + CLP group.

In order to assess whether EA at the three acupoints can regulate rat's immunity in sepsis, we detected the ratios of both CD3^+^CD4^+^ cells/CD3^+^CD8^+^ cells and Th17 cells/Treg cells in lymph node single-cell suspensions with flow cytometry (Figure 3 and 4). Groups listed below had significant differences in CD3^+^CD4^+^ cells (*F* = 125.36, *P* < 0.01), CD3^+^CD8^+^ cells (*F* = 130.806, *P* < 0.01), Th17 cells (*F* = 32.97, *P* < 0.01), and Treg cells (*F* = 146.91, *P* < 0.01) levels: Sham, CLP, CLP + Zusanli, CLP + Quchi, CLP + Tianshu, and CLP + GTS21. As reported previously, rats subjected to CLP had a downregulated level of CD3^+^CD4^+^ and Treg cells but an upregulated level of CD3^+^CD8^+^ and Th17 cells in lymph node homogenate when compared with the rats in the Sham group. Besides, the CLP + Zusanli group, along with the CLP + Quchi group, CLP + Tianshu group, and CLP + GTS21 group, had a higher proportion of CD3^+^CD4^+^ cell and a lower percentage of Treg cells than the CLP group (Figure 3 and 4). Consequently, we believed that EA at each of the three acupoints (ST36, LI11, and ST25) or administration of GTS21 could revert the immune dysfunction caused by sepsis in this CLP rat model.

### 3.5. Postsplenectomy Sepsis Rat Model Was Constructed Successfully for Mechanism Exploration

It is worth noting that EA at ST36 or at LI11 had a similar effect to the treatment of CAIP activator GTS21 in this sepsis rat model. Starting from the premises that ST36 electroacupuncture works in a CAIP way, we explored the association between ST36 stimulation and spleen. So, we established a postsplenectomy sepsis rat model, in which rats underwent splenectomy one week before the surgery of cecal ligation and puncture. Likewise, we compared the following items among the CLP group, CLP + Zusanli group, CLP + GTS21 group, SPX + CLP + Zusanli group, and SPX + CLP + GTS21 group: the 2-day survival after CLP, serum levels of TNF-*α*, IL-10, DAO, and D-LA, and the ratios of CD3^+^CD4^+^ cells/CD3^+^CD8^+^ cells and Th17 cells/Treg cells. For CLP rats that underwent splenectomy before CLP, neither EA at ST36 nor intraperitoneal injection of GTS21 can lower the 2-day death rates after CLP (Table 2.). However, the other items listed above were statistically different among those groups, with some interesting results in further comparisons.

### 3.6. EA at ST36 Alleviates Inflammatory Reaction of Septic Rats in a Spleen-Dependent Way

Compared with rats that received CLP surgery only, CLP rats undergoing splenectomy had both TNF-*α* (75.64 ± 7.60 vs. 61.91 ± 8.67 ng/mL, *P* < 0.05) and IL-10 (129.10 ± 15.41 vs. 94.07 ± 13.78 ng/mL, *P* < 0.01) increased in serum. In accordance with the result obtained in the CLP model, the use of GTS21 after CLP could also decrease the levels of both TNF-*α* (29.44 ± 9.00 vs. 75.64 ± 7.60 ng/mL, *P* < 0.01) and IL-10 (20.87 ± 4.29 vs. 129.10 ± 15.41 ng/mL, *P* < 0.01) in the CLP rats subjected to SPX. However, EA at ST36 showed no therapeutic effect on reducing the inflammatory reaction in the postsplenectomy CLP rats, as no significant difference was found between the SPX + CLP + Zusanli group and SPX + CLP group in serum TNF-*α* (66.35 ± 11.02 vs. 75.64 ± 7.60 ng/mL, *P*=0.29) and IL-10(126.59 ± 18.15 vs. 129.10 ± 15.41 ng/mL, *P*=1.00) concentration. These results (Figure 1) demonstrated that the spleen was an essential organ for ST36 electroacupuncture to alleviate inflammatory reactions in septic rats.

### 3.7. The Beneficial Effect of ST36 Electroacupuncture on Intestinal Barrier Is Partially Dependent on Spleen

As shown in Figure 2, the SPX + CLP group had a significantly higher serum level of D-LA (76.61 ± 3.40 vs. 68.90 ± 3.56 pg/mL, *P*=0.004), but not DAO (1030.56 ± 113.11 vs. 895.71 ± 126.66 pg/mL, *P* > 0.05), than the CLP group. Similarly, the SPX + CLP + Zusanli group had a lower level of D-LA (70.37 ± 3.23 vs. 76.61 ± 3.40 pg/mL, *P*=0.04) than the SPX + CLP group, but not DAO (1068.89 ± 96.51 vs. 1030.56 ± 113.11 pg/mL,*P*=0.987). Furthermore, the administration of GTS21 could reduce the secretion of DAO (555.56 ± 75.44 vs. 1030.56 ± 113.11 pg/mL, *P* < 0.01) and D-LA (35.39 ± 1.01 vs. 76.61 ± 3.40 pg/mL, *P* < 0.01) in postsplenectomy CLP rats. To sum up, the spleen participates in the protective effect of ST36 stimulation on septic rats' intestinal barrier, and there may be an alternative way involved.

### 3.8. EA at ST36 Improves the Intestinal Immunity of CLP Rats Pretreated with Splenectomy

As stated above, we observed an immunomodulatory effect of electroacupuncture at ST36 on septic rats by calculating the percentage of CD3^+^CD4^+^ cells, CD3+CD8+ cells, Th17 cells, and Treg cells in the mesenteric lymph node. In order to figure out whether this effect was associated with the spleen, we repeated this experiment on the SPX CLP rat model (Figures 3 and 4). The removal of the spleen raised the percentage of CD3^+^CD4^+^ cells (64.15 ± 0.75 vs. 65.83 ± 0.86%, *P* < 0.05) and Th17 cells (9.46 ± 0.94 vs. 7.53 ± 1.70%, *P* < 0.01) and, at the same time, lowered the percentage of CD3^+^CD8^+^ cells (38.57 ± 3.21 vs. 33.51 ± 0.66%, *P* < 0.01) and Treg cells (0.31 ± 0.45 vs. 1.09 ± 0.21%, *P* < 0.05), indicating that it may exacerbate the dysfunction of immunity. The comparison between the SPX + CLP + Zusanli group and SPX + CLP group showed that treatment of ST36 electroacupuncture reverted the mess of immunity in lymph node by enhancing the value of CD3^+^CD4^+^ cells/CD3^+^CD8^+^ cells (2.19 ± 0.04 vs. 1.67 ± 0.13%, *P* < 0.01) and Treg cells/Th17 cells (0.33 ± 0.10 vs. 0.04 ± 0.05, *P* < 0.05), just as what GTS21 administration brought to SPX rats.

## 4. Discussion

In this study, we found the following. (1) EA at LI11 or at ST36 exerted similar beneficial effects to GTS21 on the systemic inflammatory response, intestinal permeability, and intestinal T lymphocyte subsets. (2) EA at ST25 only showed the ability to modulate the intestinal T-cell component. (3). For acupuncture at ST36, the spleen is important for systemic inflammation regulation, but it is not indispensable for intestinal barrier protection and local immune defense. These results enhanced our understandings of the effects of EA on sepsis in this CLP rat model and presented a rough association between spleen and acupuncture at ST36.

Acupuncture at ST36 was effective in treating many diseases, such as ischemic bowel disease, inflammatory bowel disease, chronic obstructive pulmonary disease, and sepsis [7, 10, 29, 30]. And CAIP was found to be involved in these processes [11, 15, 31]. CAIP works dependently on acetylcholine, which bounds to AchR after being relieved from the vagus nerve, and inhibits inflammation cytokines' production and activates anti-inflammation cells. In line with previous studies, acupuncture at ST36 showed a similar anti-inflammation effect to GTS21 administration in decreasing TNF-*α* and IL-10 levels [10, 32, 33]. Moreover, the significant reduction of serum DAO and D-LA indicated that acupuncture at ST36 could protect the intestinal barrier from sepsis. In the condition of presplenectomy, acupuncture at ST36 failed to decrease the systemic inflammation (rebound in TNF-*α* and IL-10 levels) or alleviate the hypofunction in intestinal permeability (increased level of DAO). Nevertheless, the removal of the spleen could not significantly influence the anti-inflammatory effect of GTS21, probably due to its combination with *α*7nAChRs in organs like the gut, liver, kidney, and lung or associations with another anti-inflammation pathway [34–36]. Above all, the spleen is indispensable for acupuncture at ST36 to reduce the host's systemic inflammation.

Interestingly, when the spleen was removed beforehand, acupuncture at ST36 could still improve the intestinal immune function by balancing the proportion of T lymphocyte (CD3^+^CD4^+^/CD3^+^CD8^+^ cells and Treg/Th17 cells). Does it exert this function through a CAIP pathway that does not require a spleen or another pathway different from CAIP? Studies from Matteoli et al. provided some pieces of evidence for the first question [37, 38]. They reported that stimulating the vagus nerve before intestinal manipulation reduced rat's postoperative ileus rate, and this process was independent of mature T and B cells as well as spleen but required the participation of 7nAChR. Also, they showed that signal was transmitted directly to postganglionic cholinergic nerve fibers that formed a network around resident macrophages expressing 7nAChRs and, therefore, modulated immune homeostasis in the gut. To find out potential mechanisms besides CAIP, we reviewed a list of studies about acupuncture at ST36 (ST36) in experimental sepsis [39]. It is also related to the reduction of oxidative stress, suppression of the TLR4/NF-*κ*B pathway, and induction of vagus/adrenal medulla/dopamine pathway, indicating that EA at ST36 treated patients in many ways.

Notably, another focus of this study is to determine the effects of EA at LI11 and ST25 on regulating the gastrointestinal function of septic rats. Similar to ST36 treatment and GTS21 administration, EA at LI11 could decrease systemic inflammation response and intestinal permeability, while ST25 had no effects on both. These findings that EA at ST25 had different effects from ST36 and LI11 are consistent with previous studies [40, 41], indicating that its mechanism may vary from the other two. However, few studies compared the therapeutic effects of all these three acupoints, and most of them paid attention to gastrointestinal motility. As far as we know, this is the first time to observe their differences in modulating systemic inflammation and intestinal permeability. Regarding their mechanisms, it is reported that ST36 and LI11 Electrical Stimulation enhanced jejunal motility mainly through the vagus nerve (has a similar function to the parasympathetic nerve) dependent pathway and parasympathetic pathway, respectively, while ST25 inhibited jejunal motility in sympathetic pathways [42–44]. The parasympathetic and sympathetic nervous system plays an important role in neural networks to control gastrointestinal motility. By summarizing data from existing researches, Yu [45] put forward that stimulating acupoints located at arms and legs, such as ST36 and LI11, activates vagal efferent fiber and results in positive gut motility, and stimulating acupoints distributed in the abdomen (like ST25) regulates gut motility negatively in a sympathetic dependent way. Vagotomy or knockout genes encoding M2 and M3 receptors can reduce the former action and antagonist of sympathetic nerve or deletion of genes encoding *β*1 and *β*2 receptors can attenuate the latter effect [42–44, 46]. These results indicated that we can choose different acupoints for EA depending on different kinds of gastrointestinal dysfunction. Besides, further studies found that EA at ST36 and ST25 induced different connections to the central nervous system: ST36-parasympathetic pathways-dorsal motor nucleus-solitary tract nucleus and ST25-sympathetic pathways-intermediolateral column of the spinal cord-rostral ventrolateral medulla-solitary tract nucleus [45]. It provided neural anatomical evidence for us to understand the effects of acupuncture on gastric motor function in rats. However, more studies are warranted to classify its complex mechanism.

Last but not least, this study included but was not limited to the following deficiencies. (1) Although several studies supported treating sepsis through CAIP, we only used GTS21 as a positive reference when studying the association of ST36 and CAIP, and no stronger evidence was given. (2) The effect of LI11 electrostimulation is similar to that of ST36 stimulation and GST21 administration, indicating that LI11 acupuncture may have an effect via the CAIP too. However, we did not make it clear for the lack of finance. (3) We limited the study duration in 2 days after CLP. Further studies are needed to explore the long-term effect of EA.

Challenges of high incidence, high mortality, and high drug-resistance rates force us to find out a new therapeutic target to manage sepsis. Delightedly, the news of Youyou Tu and James P. Allison et al. getting the Nobile Prize subsequently did encourage us by showing the power of TCM and immunity in dealing with diseases. The creative practice of TCM based on deep understanding may help us to relieve the burdens of sepsis prevention and treatment.

## 5. Conclusion

The results of this study indicate that EA at ST36 or LI11 had similar effects on modulating systemic inflammatory response, intestinal permeability, and intestinal T-cell component of CLP rats compared to GTS21, while EA at ST25 only showed improvement in intestinal T lymphocyte. Spleen might be essential for the regulation of systemic inflammation in acupuncture at ST36 but not indispensable for the modulation of the intestinal barrier and immune defense. Electroacupuncture might be a potential therapy to regulate inflammation and immunity in septic patients.

## Figures and Tables

**Table 1:** The 2-day mortality of CLP rats in each group.

Groups	Total number	Deaths	Number of rats included in each analysis	Mortality
CLP	12	5	7	41.67
CLP + Zusanli	12	5	7	41.67
CLP + Quchi	12	7	5	58.33
CLP + Tianshu	12	5	7	41.67
CLP + GTS21	12	7	5	58.33

**Table 2:** The 2-day death rate of postsplenectomy CLP rats.

Groups	Total number	Deaths	Number of rats included in each analysis	Mortality (%)
SPX + CLP	12	6	6	50.00
SPX + CLP + Zusanli	12	6	6	50.00
SPX + CLP + GTS21	12	6	6	50.00

## Data Availability

The datasets used and/or analyzed during the current study are available from the corresponding author on reasonable request.

## Abbreviations

EA: Electroacupuncture
CAIP: Cholinergic Anti-Inflammatory Pathway
CLP: Cecal ligation and puncture
SPX: Postsplenectomy
*α*7nAchR: *α*7 Nicotinic acetylcholine receptor
D-LA: D-lactic acidosis
DAO: Double amine oxidase
TCM: Traditional Chinese Medicine.

## Supplementary Materials

Supplementary Materials Supplemental Table 1: the modulation effects on the plasma TNF-*α*, IL-10, DAO, and D-LA expression in CLP rats. Supplemental Table 2: the modulation effects on the CD3^+^CD4^+^/CD3^+^CD8^+^ levels in the intestinal lymph node in CLP rats. Supplemental Table 3: the modulation effects on Treg/Th17 levels in the intestinal lymph node in CLP rats. Supplemental Table 4: per-splenectomy takes part in the modulating process of Zusanli electroacupuncture on the plasma TNF-*α*, IL-10, DAO, and D-LA expression in CLP rats. Supplemental Table 5: spleen is not essential for acupuncture at ST36 in regulating the CD3^+^CD4^+^/CD3^+^CD8^+^ proportion in the intestinal lymph node. Supplemental Table 6: the effect of acupuncture at ST36 adjusting the ratio of Treg/Th17 in the intestinal lymph node is not dependent on the spleen. Supplemental Figure 1: CD3^+^CD4^+^ and CD3^+^CD8^+^ proportion in intestinal lymph node in CLP rats. Supplemental Figure 2: Th17 cells and Treg proportion in the intestinal lymph node in CLP rats. Supplemental Figure 3(a): CD3+CD4+ proportion in the intestinal lymph node in CLP rats and SPX + CLP rats. Supplemental Figure 3(b): CD3^+^CD8^+^ proportion in the intestinal lymph node in CLP rats and SPX + CLP rats. Supplemental Figure 4(a): Foxp3 proportion in the intestinal lymph node in CLP rats and SPX + CLP rats. Supplemental Figure 4(b): Th17 proportion in the intestinal lymph node in CLP rats and SPX + CLP rats.
