# Supplementary material for: Effect of Electroacupuncture at Zusanli (ST36) on Sepsis Induced by Cecal Ligation Puncture and Its Relevance to Spleen
Source: Evid Based Complement Alternat Med. 2020 Oct 7;2020:1914031. doi: 10.1155/2020/1914031 (PMC7563055; doi:10.1155/2020/1914031)
Supplement: Supplementary Materials — Supplemental Table 1: the modulation effects on the plasma TNF-α, IL-10, DAO, and D-LA expression in CLP rats. Supplemental Table 2: the modulation effects on the CD3+CD4+/CD3+CD8+ levels in the intestinal lymph node in CLP rats. Supplemental Table 3: the modulation effects on Treg/Th17 levels in the intestinal lymph node in CLP rats. Supplemental Table 4: per-splenectomy takes part in the modulating process of Zusanli electroacupuncture on the plasma TNF-α, IL-10, DAO, and D-LA expression in CLP rats. Supplemental Table 5: spleen is not essential for acupuncture at ST36 in regulating the CD3+CD4+/CD3+CD8+ proportion in the intestinal lymph node. Supplemental Table 6: the effect of acupuncture at ST36 adjusting the ratio of Treg/Th17 in the intestinal lymph node is not dependent on the spleen. Supplemental Figure 1: CD3+CD4+ and CD3+CD8+ proportion in intestinal lymph node in CLP rats. Supplemental Figure 2: Th17 cells and Treg proportion in the intestinal lymph node in CLP rats. Supplemental Figure 3(a): CD3+CD4+ proportion in the intestinal lymph node in CLP rats and SPX + CLP rats. Supplemental Figure 3(b): CD3+CD8+ proportion in the intestinal lymph node in CLP rats and SPX + CLP rats. Supplemental Figure 4(a): Foxp3 proportion in the intestinal lymph node in CLP rats and SPX + CLP rats. Supplemental Figure 4(b): Th17 proportion in the intestinal lymph node in CLP rats and SPX + CLP rats. [file 1914031.f1.doc]

**Supplemental Table 1. The modulation eﬀects on plasma TNF-α, IL-10, DAO, and D-LA expression in CLP rats (mean ± SD).**

| Groups | Cytokines(ng/mL) | | Exudates(pg/mL) | |  |
| --- | --- | --- | --- | --- | --- |
|  | TNF-α | IL-10 | DAO | D-LA | |
| Sham | 23.27±1.96^**^ | 18.01±2.25^**^ | 177.22±34.35^**^ | 28.31±3.63^**^ | |
| CLP | 61.91±8.67 | 94.07±13.78 | 895.71±126.66 | 68.90±3.56 | |
| CLP + Zusanli | 31.27±3.53^**^ | 71.16±15.75^*^ | 492.86±39.51^**^ | 39.54±2.72^**^ | |
| CLP + Quchi | 32.92±2.24^**^ | 63.87±9.27^**^ | 510.00±19.15^**^ | 39.78±1.60^**^ | |
| CLP + Tianshu | 56.13±8.92 ^NS^ | 108.96±15.43 ^NS^ | 940.00±103.03 ^NS^ | 64.70±7.16 ^NS^ | |
| CLP + GTS21 | 29.44±9.00^**^ | 20.87±4.29^**^ | 378.67±73.20^**^ | 33.32±5.16^**^ |  |
| *F* | 37.17 | 58.76 | 89.36 | 93.68 |  |
| *P* | ﹤0.001 | ﹤0.001 | ﹤0.001 | ﹤0.001 |  |

Compared with CLP group: ^NS^- no significant different; ^*^-*P*＜0.05；^**^-*P*＜0.01.

**Supplemental Table 2. The modulation eﬀects on CD3^+^CD4^+^/ CD3^+^CD8^+^ levels in intestinal lymph node in CLP rats (mean ± SD).**

| Groups | Number | CD3+CD4+(%) | CD3^+^CD8^+^(%) | CD3+CD4+/  CD3+CD8+ |
| --- | --- | --- | --- | --- |
| Sham | 6 | 75.78±0.58** | 21.88±0.93** | 3.47±0.13** |
| CLP | 7 | 65.83±0.86 | 33.51±0.66 | 1.96±0.05 |
| CLP + Zusanli | 7 | 72.73±0.98** | 25.76±1.09** | 2.83±0.09** |
| CLP + Quchi | 5 | 71.38±0.70** | 27.72±1.24** | 2.58±0.12** |
| CLP + Tianshu | 7 | 73.54±1.18** | 24.47±0.83** | 3.01±0.09** |
| CLP + GTS21 | 5 | 78.92±1.34** | 19.82±1.56** | 4.01±0.37** |
| *F* |  | 125.36 | 130.80 | 112.26 |
| *P* |  | ＜0.001 | ＜0.001 | ＜0.001 |

Compared with CLP group: ^NS^- no significant different; ^*^-*P*＜0.05；^**^-*P*＜0.01.

**Supplemental Table 3. The modulation eﬀects on Treg/Th17 levels in intestinal lymph node in CLP rats (mean ± SD).**

| Groups | n | Treg(%) | Th17(%) | Treg/Th17 |
| --- | --- | --- | --- | --- |
| Sham | 6 | 4.81±0.64** | 2.62±0.14** | 1.84±0.29** |
| CLP | 7 | 1.09±0.21 | 7.53±1.70 | 0.15±0.05 |
| CLP + Zusanli | 7 | 2.99±0.25** | 3.48±0.24** | 0.86±0.09** |
| CLP + Quchi | 5 | 2.34±0.24** | 3.84±1.08** | 0.65±0.18** |
| CLP + Tianshu | 7 | 3.57±0.22** | 3.20±0.19** | 1.12±0.09** |
| CLP + GTS21 | 5 | 6.67±0.59** | 2.09±0.27** | 3.21±0.31** |
| *F* |  | 146.91 | 32.97 | 194.07 |
| *P* |  | ﹤0.001 | ﹤0.001 | ﹤0.001 |

Compared with CLP group: ^NS^- no significant different; ^*^-*P*＜0.05；^**^-*P*＜0.01.

**Supplemental Table 4. Per-splenectomy takes part in the modulating process of Zusanli electro-acupuncture on plasma TNF-α, IL-10, DAO, and D-LA expression in CLP rats (mean ± SD).**

| Groups | Cytokines(ng/mL) | | Exudates(pg/mL) | | |  |
| --- | --- | --- | --- | --- | --- | --- |
|  | TNF-α | IL-10 | | DAO | D-LA | |
| Sham | 23.27±1.96^##^ | 18.01±2.25^##^ | | 177.22±34.35^##^ | 28.31±3.63^##^ | |
| CLP | 61.91±8.67^#^ | 94.07±13.78^##^ | | 895.71±126.66^ns^ | 68.90±3.56^##^ | |
| CLP + Zusanli | 31.27±3.53^##^ | 71.16±15.75^##^ | | 492.86±39.51^##^ | 39.54±2.72^##^ | |
| CLP + GTS21 | 29.44±9.00^##^ | 20.87±4.29^##^ | | 378.67±73.20^##^ | 33.32±5.16^##^ | |
| SPX + CLP | 75.64±7.60 | 129.10±15.41 | | 1030.56±113.11 | 76.61±3.40 | |
| SPX + CLP + Zusanli | 66.35±11.02^ns^ | 126.59±18.15^ns^ | | 1068.89±96.51^ns^ | 70.37±3.23^#^ | |
| SPX + CLP + GTS21 | 30.73±3.62^##^ | 42.98±10.29^##^ | | 555.56±75.44^##^ | 35.39±1.01^##^ | |
| *F* | 55.45 | 75.24 | | 94.21 | 227.62 | |
| *P* | ﹤0.001 | ﹤0.001 | | ﹤0.001 | ﹤0.001 | |

Compared with SPX+CLP group: ^ns^- no significant different; ^#^-P＜0.05；^##^-P＜0.01.

**Supplemental Table 5. Spleen is not essential for Zusanli acupuncture therapy in regulating CD3^+^CD4^+^/ CD3^+^CD8^+^ proportion in intestinal lymph node.**

| Groups | Number | CD3^+^CD4^+^(%) | CD3+CD8+(%) | CD3+CD4+/ CD3+CD8+ |
| --- | --- | --- | --- | --- |
| Sham | 6 | 75.78±0.58^##^ | 21.88±0.93^##^ | 3.46±0.13^##^ |
| CLP | 7 | 65.83±0.86^#^ | 33.51±0.66^##^ | 1.96±0.05^#^ |
| CLP + Zusanli | 7 | 72.73±0.98^##^ | 25.76±1.09^##^ | 2.83±0.09^##^ |
| CLP + GTS21 | 5 | 78.92±1.34^##^ | 19.82±1.56^##^ | 4.01±0.37^##^ |
| SPX + CLP | 6 | 64.15±0.75 | 38.57±3.21 | 1.67±0.13 |
| SPX + CLP + Zusanli | 6 | 68.43±0.71^##^ | 31.25±0.59^##^ | 2.19±0.04^##^ |
| SPX + CLP + GTS21 | 6 | 68.95±0.58^##^ | 31.71±0.58^##^ | 2.17±0.04^##^ |
| *F* |  | 228.15 | 119.09 | 185.35 |
| *P* |  | ﹤0.001 | ﹤0.001 | ﹤0.001 |

Compared with SPX+CLP group: ^ns^- no significant different; ^#^-P＜0.05；^##^-P＜0.01.

**Supplemental Table 6. The effect of Zusanli acupuncture therapy that adjusting the ratio of Treg/Th17 in intestinal lymph node is not dependent on spleen.**

| Groups | Number | Treg(%) | Th17(%) | Treg/Th17 |
| --- | --- | --- | --- | --- |
| Sham | 6 | 4.81±0.64^##^ | 2.62±0.14^##^ | 1.84±0.29^##^ |
| CLP | 7 | 1.09±0.21^#^ | 7.53±1.70^##^ | 0.15±0.05^ns^ |
| CLP + Zusanli | 7 | 2.99±0.25^##^ | 3.48±0.24^##^ | 0.86±0.09^##^ |
| CLP + GTS21 | 5 | 6.67±0.59^##^ | 2.09±0.27^##^ | 3.21±0.31^##^ |
| SPX + CLP | 6 | 0.31±0.45 | 9.46±0.94 | 0.04±0.05 |
| SPX + CLP + Zusanli | 6 | 1.60±0.43^##^ | 4.87±0.46^##^ | 0.33±0.10^#^ |
| SPX + CLP + GTS21 | 6 | 1.88±0.20^##^ | 4.69±0.79^##^ | 0.41±0.09^##^ |
| $\chi^{2}$ |  | 163.30 | 57.43 | 271.22 |
| *P* |  | ﹤0.001 | ﹤0.001 | ﹤0.001 |

Compared with SPX+CLP group: ^ns^- no significant different; ^#^-P＜0.05；^##^-P＜0.01.

**Supplemental Figure 1. CD3+CD4+ and CD3+CD8+ proportion in intestinal lymph node in CLP rats.**

**A.**

CLP + GTS21

Sham

CLP

CLP + Zusanli

CLP + Quchi

CLP + Tianshu

Sham

CLP

CLP + Zusanli

**B.**

CLP + Quchi

CLP + GTS21

CLP + Tianshu

**Figure 4. (A & B)**

**Supplemental Figure 2. Th17** **cells and Treg proportion in intestinal lymph node in CLP rats.**

Sham

CLP

CLP + Zusanli

**A.**

CLP + Quchi

CLP + Tianshu

CLP + GTS21

Sham

CLP

CLP + Zusanli

**B.**

CLP + Tianshu

CLP + GTS21

CLP + Quchi

**Supplemental Figure 3A. CD3+CD4+ proportion in intestinal lymph node in CLP rats and SPX+CLP rats.**

Sham

CLPP

CLP + Zusanli

CLP

CLP + GTS21

SPX + CLP

SPX + CLP + Zusanli

SPX + CLP + GTS21

**Supplemental Figure 3B. CD3+CD8+ proportion in intestinal lymph node in CLP rats and SPX+CLP rats.**

Sham

CLPP

CLP + Zusanli

CLP + GTS21

SPX + CLP

SPX + CLP + Zusanli

SPX + CLP + GTS21

**Supplemental Figure 4A. Foxp3 proportion in intestinal lymph node in CLP rats and SPX+CLP rats.**

Sham

CLPP

CLP + Zusanli

CLP + GTS21

SPX + CLP

SPX + CLP + Zusanli

SPX + CLP + GTS21

**Supplemental Figure 4B. Th17 proportion in intestinal lymph node in CLP rats and SPX+CLP rats.**

Sham

CLPP

CLP + Zusanli

CLP + GTS21

SPX + CLP

SPX + CLP + Zusanli

SPX + CLP + GTS21
